# Draft Genome Sequence of the Radioresistant Bacterium Deinococcus aerius TR0125, Isolated from the High Atmosphere above Japan

**DOI:** 10.1128/genomeA.00080-18

**Published:** 2018-03-01

**Authors:** Katsuya Satoh, Hiroki Arai, Toshihiko Sanzen, Yuko Kawaguchi, Hidenori Hayashi, Shin-ichi Yokobori, Akihiko Yamagishi, Yutaka Oono, Issay Narumi

**Affiliations:** a Department of Radiation-Applied Biology Research, Takasaki Advanced Radiation Research Institute, Quantum Beam Science Research Directorate, National Institutes for Quantum and Radiological Science and Technology, Takasaki, Gunma, Japan; b Faculty of Engineering, Maebashi Institute of Technology, Maebashi, Gunma, Japan; c Faculty of Life Sciences, Toyo University, Itakura, Gunma, Japan; d Department of Applied Life Sciences, Tokyo University of Pharmacy and Life Sciences, Hachioji, Tokyo, Japan; e Graduate School of Life Sciences, Toyo University, Itakura, Gunma, Japan

## Abstract

Deinococcus aerius strain TR0125 is a bacterium isolated from the high atmosphere above Japan that shows strong resistance to desiccation, UV-C, and gamma radiation. Here, we report the draft genome sequence of D. aerius (4.5 Mb), which may provide useful genetic information supporting its biochemical features.

## GENOME ANNOUNCEMENT

Members of the genus Deinococcus are best known as radioresistant bacteria, and about 70 Deinococcus species have been isolated from various environments over 60 years (http://www.bacterio.net/deinococcus.html) since the first isolation of Deinococcus radiodurans (1). Recently, the whole-genome sequences of some Deinococcus bacteria were determined. These provide important information to elucidate the unique features of Deinococcus bacteria. Deinococcus aerius strain TR0125 was initially isolated as an orange-pigmented, nonmotile, desiccation-tolerant, UV- and gamma-resistant, and coccoid bacterium from the upper troposphere in Japan (2–4).

The draft genome sequence of D. aerius strain TR0125 had a total length of 4,524,446 bp, with 50-fold average coverage and an average G+C content of 68.0%, and it comprised 43 large contigs (>500 bp). The sequences obtained with the Roche GS FLX+ and GS Junior+ systems were assembled using the GS *De Novo* Assembler version 3.0. Automatic annotation was performed using the Microbial Genome Annotation Pipeline (5), which predicted a total of 4,446 protein-coding sequences (CDSs). Moreover, all CDSs were manually validated. The tRNA and rRNA operon (5S/16S/23S) detection was performed using the tRNAscan software version 1.23 (6) and RNAmmer software version 1.2 (7), which predicted a total of 52 tRNAs and 1 rRNA operon, respectively.

Since D. aerius strain TR0125 was negative for nitrate reduction, urease, arginine hydrolase, and ornithine decarboxylase activities and was unable to use some carbon sources (3), the genes regarding these biochemical features were searched. The annotation of the draft genome sequence indicates that strain TR0125 does not possess some genes (*narB*, *ureAB*, *ureC*, *arcA*, *nosA*, *nylA*, *speC*, *speF*, *gpmA,* and *betB*) involved in nitrogen, arginine, ornithine, and carbohydrate metabolism. Strain TR0125 exhibited much slower growth than some Deinococcus species (e.g., D. radiodurans, D. grandis, and D. geothermalis) (4). This feature might be related to the fact that the strain TR0125 genome possesses only one rRNA operon as an important component for protein synthesis, although many Deinococcus bacteria possess multiple rRNA operons (8, 9). The relationship between the number of rRNA operons and the cell growth rate in other bacteria, such as Bacillus subtilis and Escherichia coli, has also been reported (10, 11). Like some Deinococcus species (e.g., D. deserti, D. gobiensis, and D. maricopensis, but excluding D. radiodurans, D. grandis, and D. geothermalis), the strain TR0125 genome encodes a DNA photolyase involved in UV resistance. Naturally, strain TR0125 possesses genes (including *pprI*, *pprA*, *recA*, *ddrA*, and *ddrO*) regarding the radiation/desiccation response system (12), which is the best-known unique feature of Deinococcus bacteria. In the future, the draft genome sequence of D. aerius strain TR0125 will be useful to elucidate the common principles of its radioresistance based on the highly efficient DNA repair mechanisms in Deinococcus bacteria by the comparative analysis of genomic sequences.

### Accession number(s).

The draft genome sequence of D. aerius strain TR0125 was deposited at DDBJ/EMBL/GenBank under the accession number BFAG00000000. The version described in this paper is the first version, BFAG01000000.

## References

[1] Anderson AW, Nordan HC, Cain RF, Parrish G, Duggan D 1956 Studies on a radioresistant *Micrococcus*. I. Isolation, morphology, cultural characteristics, and resistance to gamma radiation. Food Technol 10:575–578.

[2] Yang Y, Itahashi S, Yokobori S, Yamagishi A 2008 UV-resistant bacteria isolated from upper troposphere and lower stratosphere. Biol Sci Space 22:18–25. doi:10.2187/bss.22.18.

[3] Yang Y, Itoh T, Yokobori S, Itahashi S, Shimada H, Satoh K, Ohba H, Narumi I, Yamagishi A 2009 *Deinococcus aerius* sp. nov., isolated from the high atmosphere. Intl J Syst Evol Microbiol 59:1862–1866. doi:10.1099/ijs.0.007963-0. 19567578

[4] Kawaguchi Y, Yang Y, Kawashiri N, Shiraishi K, Takasu M, Narumi I, Satoh K, Hashimoto H, Nakagawa K, Tanigawa Y, Momoki YH, Tanabe M, Sugino T, Takahashi Y, Shimizu Y, Yoshida S, Kobayashi K, Yokobori S, Yamagishi A 2013 The possible interplanetary transfer of microbes: assessing the viability of *Deinococcus* spp. under the ISS environmental conditions for performing exposure experiments of microbes in the Tanpopo mission. Orig Life Evol Biosph 43:411–428. doi:10.1007/s11084-013-9346-1. 24132659

[5] Sugawara H, Ohyama A, Mori H, Kurokawa K 2009 Microbial genome annotation pipeline (MiGAP) for diverse users, abstr S-001, p 1–2. Abstr 20th Int Conf Genome Informatics, Kanagawa, Japan.

[6] Schattner P, Brooks AN, Lowe TM 2005 The tRNAscan-SE, snoscan and snoGPS web servers for the detection of tRNAs and snoRNAs. Nucleic Acids Res 33:W686–W689. doi:10.1093/nar/gki366. 15980563 PMC1160127

[7] Lagesen K, Hallin P, Rødland EA, Staerfeldt HH, Rognes T, Ussery DW 2007 RNAmmer: consistent and rapid annotation of ribosomal RNA genes. Nucleic Acids Res 35:3100–3108. doi:10.1093/nar/gkm160. 17452365 PMC1888812

[8] Yuan M, Chen M, Zhang W, Lu W, Wang J, Yang M, Zhao P, Tang R, Li X, Hao Y, Zhou Z, Zhan Y, Yu H, Teng C, Yan Y, Ping S, Wang Y, Lin M 2012 Genome sequence and transcriptome analysis of the radioresistant bacterium *Deinococcus gobiensis*: insights into the extreme environmental adaptations. PLoS One 7:e34458. doi:10.1371/journal.pone.0034458. 22470573 PMC3314630

[9] Satoh K, Onodera T, Omoso K, Takeda-Yano K, Katayama T, Oono Y, Narumi I 2016 Draft genome sequence of the radioresistant bacterium *Deinococcus grandis*, isolated from freshwater fish in Japan. Genome Announc 4:e01631-15. doi:10.1128/genomeA.01631-15. 26868384 PMC4751308

[10] Nanamiya H, Sato M, Masuda K, Sato M, Wada T, Suzuki S, Natori Y, Katano M, Akanuma G, Kawamura F 2010 *Bacillus subtilis* mutants harbouring a single copy of the rRNA operon exhibit severe defects in growth and sporulation. Microbiology 156:2944–2952. doi:10.1099/mic.0.035295-0. 20634236

[11] Gyorfy Z, Draskovits G, Vernyik V, Blattner FF, Gaal T, Posfai G 2015 Engineered ribosomal RNA operon copy-number variants of *E. coli* reveal the evolutionary trade-offs shaping rRNA operon number. Nucleic Acids Res 43:1783–1794. doi:10.1093/nar/gkv040. 25618851 PMC4330394

[12] Ishino Y, Narumi I 2015 DNA repair in hyperthermophilic and hyperradioresistant microorganisms. Curr Opin Microbiol 25:103–112. doi:10.1016/j.mib.2015.05.010. 26056771

